# Neurotransmitter alterations in embryonic succinate semialdehyde dehydrogenase (SSADH) deficiency suggest a heightened excitatory state during development

**DOI:** 10.1186/1471-213X-8-112

**Published:** 2008-11-28

**Authors:** Erwin EW Jansen, Eduard Struys, Cornelis Jakobs, Elizabeth Hager, O Carter Snead, K Michael Gibson

**Affiliations:** 1 Metabolic Unit, Department of Clinical Chemistry, VU Medical Center, Amsterdam, the Netherlands; 2 Division of Medical Genetics, Department of Pediatrics, Children's Hospital & the University of Pittsburgh School of Medicine, Pittsburgh, PA, USA; 3 Division of Pediatric Neurology, Brain and Behavior Institute, Hospital for Sick Children & the University of Toronto, Toronto, Ontario, Canada; 4 Department of Pathology, Biochemical Genetics Laboratory, University of Pittsburgh Medical Center & the University of Pittsburgh School of Medicine, Pittsburgh, PA, USA; 5 Department of Human Genetics, Graduate School of Public Health, University of Pittsburgh Medical Center & the University of Pittsburgh School of Medicine, Pittsburgh, PA, USA

## Abstract

**Background:**

SSADH (aldehyde dehydrogenase 5a1 (Aldh5a1); γ-hydroxybutyric (GHB) aciduria) deficiency is a defect of GABA degradation in which the neuromodulators GABA and GHB accumulate. The human phenotype is that of nonprogressive encephalopathy with prominent bilateral discoloration of the globi pallidi and variable seizures, the latter displayed prominently in Aldh5a1^-/- ^mice with lethal convulsions. Metabolic studies in murine neural tissue have revealed elevated GABA [and its derivatives succinate semialdehyde (SSA), homocarnosine (HC), 4,5-dihydroxyhexanoic acid (DHHA) and guanidinobutyrate (GB)] and GHB [and its analogue D-2-hydroxyglutarate (D-2-HG)] at birth. Because of early onset seizures and the neurostructural anomalies observed in patients, we examined metabolite features during Aldh5a1^-/- ^embryo development.

**Methods:**

Embryos were obtained from pregnant dams sacrificed at E (embryo day of life) 10–13, 14–15, 16–17, 18–19 and newborn mice. Intact embryos were extracted and metabolites quantified by isotope dilution mass spectrometry (n = 5–15 subjects, Aldh5a1^+/+ ^and Aldh5a1^-/-^) for each gestational age group. Data was evaluated using the *t *test and one-way ANOVA with Tukey post hoc analysis. Significance was set at the 95^th ^centile.

**Results:**

GABA and DHHA were significantly elevated at all gestational ages in Aldh5a1^-/- ^mice, while GB was increased only late in gestation; SSA was not elevated at any time point. GHB and D-2-HG increased in an approximately linear fashion with gestational age. Correlative studies in human amniotic fluid from SSADH-deficient pregnancies (n = 5) also revealed significantly increased GABA.

**Conclusion:**

Our findings indicate early GABAergic alterations in Aldh5a1^-/- ^mice, possibly exacerbated by other metabolites, which likely induce a heightened excitatory state that may predispose neural networks to epilepsy in these animals.

## Background

**Figure 1:**
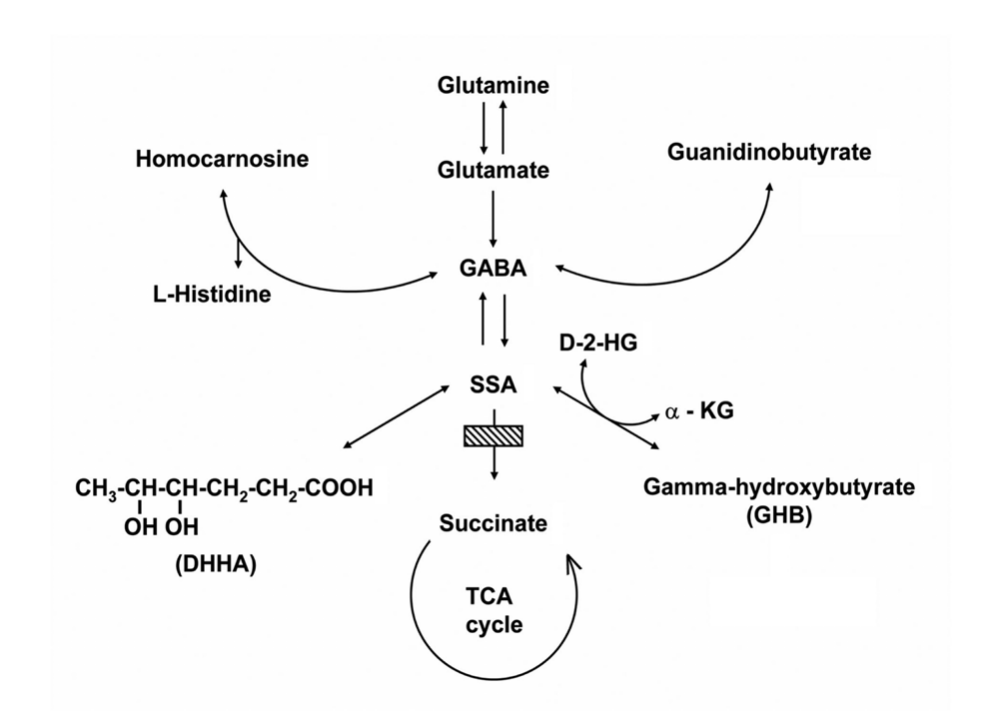
**Schematic of GABA degradation and metabolites accumulating (arrows adjacent to metabolites) in Aldh5a1 deficiency (depicted by cross-hatched box).** Abbreviations: SSA, succinate semialdehyde; α-KG, α-ketoglutarate; D-2-HG, D-2-hydroxyglutarate; DHHA, 4,5-dihydroxyhexanoic acid; TCA, tricarboxylic acid.

Succinic semialdehyde dehydrogenase (SSADH) deficiency (aldehyde dehydrogenase 5a1 (Aldh5a1); E.C. 1.2.1.24; OMIM 271980, 610045) is a rare neurometabolic defect of the GABA catabolic pathway (Fig. 1). The phenotype is variably that of a static encephalopathy, associated with developmental delays, hypotonia, ataxia, defects or absence of speech, and seizures [1]. Older patients may demonstrate a prominent component of neuropsychiatric morbidity. A cardinal finding from imaging of patients is hyper-intense signals in the globi pallidi bilaterally [2]. Identification of affected probands is facilitated by detection of increased γ-hydroxybutyric acid (GHB) during routine urine organic acid analysis. GHB, a by-product of defective GABA catabolism (Fig. 1), is a compound with its own unique pharmacological profile, and it remains a topic of debate as to whether it is a neurotransmitter as is the case for its parent compound, GABA [3]. Treatment options for patients are limited and primarily palliative, although recent findings in mice suggest that a ketogenic diet may have therapeutic relevance [4,5].

In an effort to examine the pathophysiology of SSADH deficiency and explore effective preclinical treatment paradigms, Hogema and coworkers [6] developed a murine knockout model. While lethality does not occur in the early newborn period, this animal demonstrates almost uniform lethality in status epilepticus at about 3–4 weeks of life, associated with a marked failure to thrive. Neurophysiological evaluations have revealed a seizure transition process that progresses from absence to generalized tonic-clonic convulsions [7], associated with marked dysregulation of GABAergic neurotransmission in the presence of essentially normal glutamatergic and GHBergic function [7-10]. Pharmacotherapeutic approaches to offset early lethality have been described above, but also have included the GHB receptor antagonist NCS-382, the GABA_B _receptor antagonist CGP 35348, and the irreversible GABA transaminase inhibitor vigabatrin (Fig. 1), the latter perhaps the most widely invoked pharmacotherapy in this disorder, despite potential side-effects [11,12].

Previous studies from our laboratory have shown that significant elevations of GHB and GABA occur in newborn Aldh5a1^-/- ^mice, and these persist until premature death [13]. Elevations of GHB were accompanied by a progressive decrease in glutamine levels with age, which is of interest since glutamine serves as a key shuttle molecule maintaining astrocytic and neuronal concentrations of glutamate and GABA [14] (Fig. 1). GABA, the GHB precursor, was also significantly elevated in the newborn Aldh5a1^-/- ^mice [13]. Based upon these accumulations, and in view of the seizure phenotype of Aldh5a1^-/- ^mice and the imaging abnormalities observed in human patients, we hypothesized that GABA might be significantly elevated in embryonic Aldh5a1^-/- ^mice. The relevance of this question is underscored by the observation that early in rodent embryonic development, and through the first week of postnatal life, GABA exerts excitatory actions that do not transition to inhibitory potentials until chloride concentrations level out by approximately postnatal week 2 [15,16]. The current report summarizes our metabolic characterization of Aldh5a1 embryos, and provides evidence that a heightened excitatory state may exist in Aldh5a1^-/- ^mice during early development.

## Methods

Husbandry and PCR-genotyping of the Aldh5a1 mutant mouse colony has been previously described [17]. Mutant mice were generated by heterozygous matings (Aldh5a1^+/- ^× Aldh5a1^+/-^); animals were C57Bl/6 congenics. All procedures involving mice were approved by the Institutional Animal Research Care Committee (ARCC), and were performed in accordance with NIH guidelines for laboratory animals.

Timed matings were established for embryo generation. During daily checking, the identification of a vaginal plug in the dams was set at E6.5, and pregnancies were allowed to continue to predetermined timepoints. Dams were rapidly sacrificed and the embryos dissected on a cold-plate (4°C), being careful to avoid maternal decidua. Embryos were either prepared immediately or stored at -80°C until further work-up. Because dissection of embryos was not feasible, all were identically handled without dissection of neural tissue. Additionally, it might have been optimal to isolate enriched neural tissue from these embryos (e.g., brain). However, GABA exerts activities (and is found in measurable quantities) in peripheral tissues, including liver, kidney and pancreas, and thus in these studies we sought to obtain the most complete picture of GABA (and related metabolites) in these embryos [18]. After weighing, a powder of each embryo was prepared by rapid pulverization in dry-ice/acetone. The powders were reconstituted using phosphate-buffered saline (pH 7.4) at a constant ratio of 0.2 g tissue/ml, extracted with a Dounce homogenizer on ice, and finally clarified by centrifugation. The supernatants were stored for metabolite quantification at -80°C; protein content was determined using the Bradford method with BSA as standard. To achieve a statistically significant number in each group, embryos were grouped as follows: E10-13, E14-15, E16-17, E18-19 and P (postnatal)1. For each group (Aldh5a1^+/+^, Aldh5a1^-/-^), n = 5–15 separate subjects.

GABA, D- and L-2-hydroxyglutaric acids (D-2-HG/L-2-HG), SSA, DHHA, GHB and guanidinobutyrate (GB) were quantified by isotope dilution methodology employing either gas chromatography-mass spectrometry or liquid chromatography-tandem mass spectrometry (LC/MS-MS) [17,19-23]. The same metabolites were quantified in amniotic fluid specimens submitted for prenatal diagnosis in pregnancies at-risk for Aldh5a1 deficiency. In the amniotic fluids characterized, the subsequent propositus was identified as Aldh5a1-deficient either via enzyme or mutation analysis in blood, or both. Amino acids in clarified supernatants were quantified by HPLC analysis with ninhydrin post-column detection, using established methods [13]. Data was evaluated using the GraphPad Prizm program (version 4.0) using the *t *test and one-way ANOVA with Tukey post hoc analysis. Significance was set at the 95^th ^centile.

## Results

**Figure 2:**
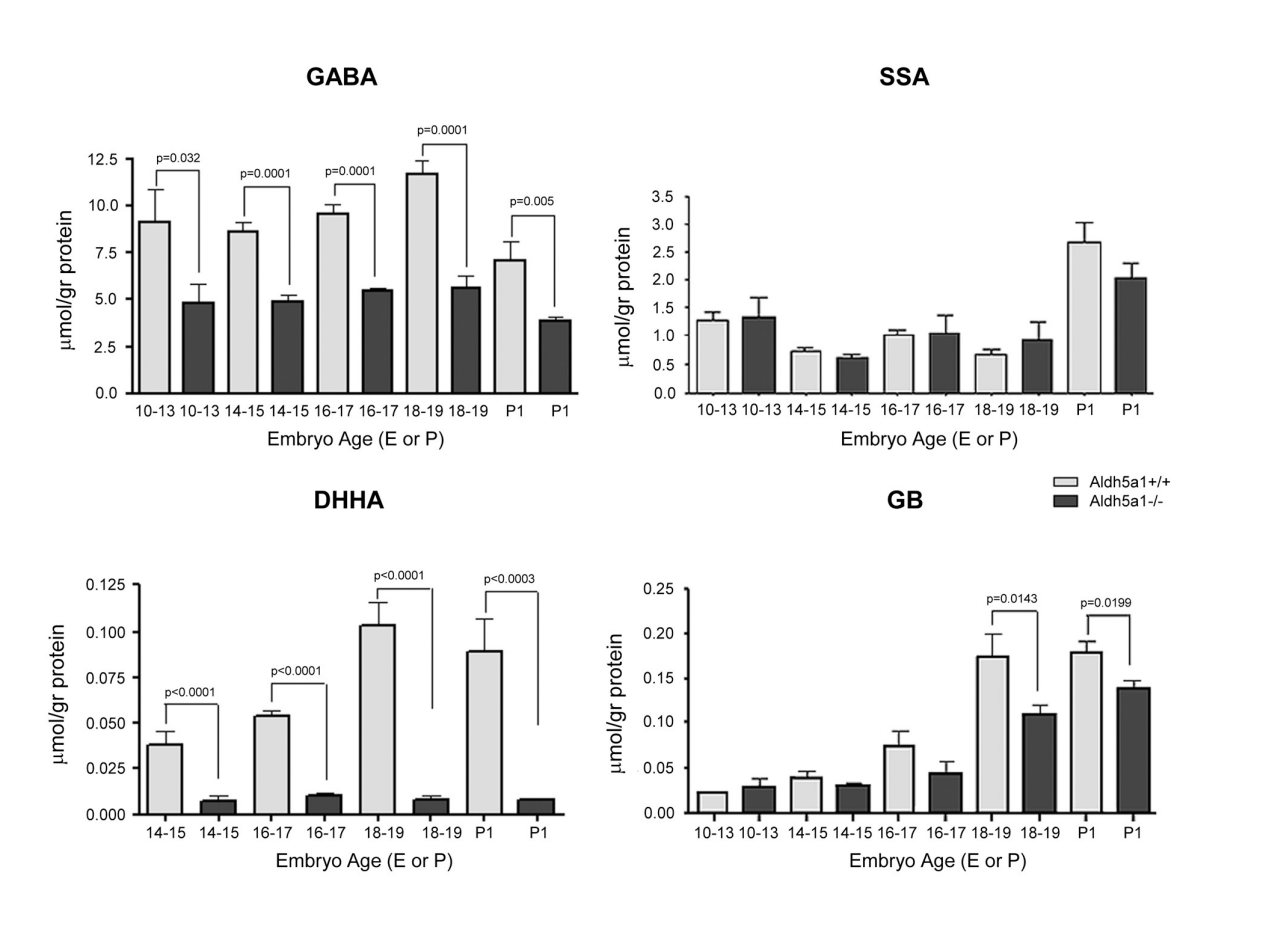
**Concentrations of GABA, DHHA (4,5-dihydroxyhexanoic acid), SSA (succinate semialdehyde) and GB (guanidinobutyrate) in embryo extracts as a function of developmental age.** Statistical analysis performed using a two-tailed t test. Abbreviations: gr, gram; E, embryonic (for example, 10–13 is equivalent to E10–E13); P, postnatal. Note that E 10–13 embryo extracts were not available for measurement of DHHA.

**Figure 3:**
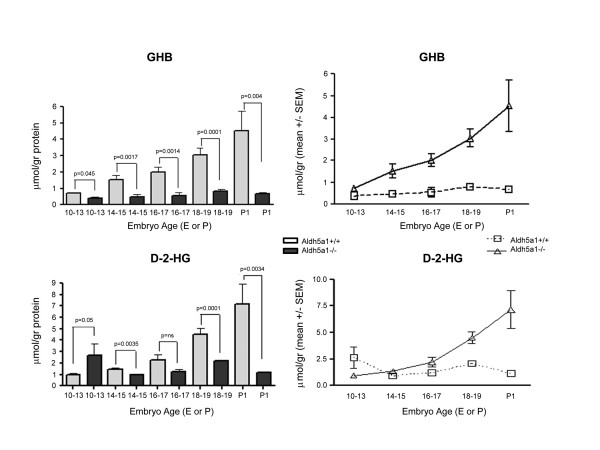
**Concentrations of GHB (gamma-hydroxybutyric acid) and D-2-HG (D-2-hydroxyglutarate) in embryo extracts as a function of age.** A linear representation of means ± SEM by genotype is depicted as well. Abbreviations and statistical analysis as described in Fig. 2. SEM, standard error of the mean.

Relevant metabolite findings in embryos of both genotypes (Aldh5a1^+/+^, Aldh5a1^-/-^) are described in Figs. 2 and 3. GABA was significantly increased at all embryonic ages as a function of genotype (Fig. 2). The concentration by genotype was roughly constant during embryonic development, although a drop in GABA concentrations at birth in both genotypes failed to reach statistical significance (one-way ANOVA with Tukey post-hoc analysis). The concentration of the GABA transamination reaction product, succinic semialdehyde (SSA), was not different by genotype at any gestational timepoint, implying an efficient conversion of SSA to other intermediates (e.g., DHHA, GHB). As noted in Fig. 3, however, there was considerable variation of SSA with genotype, yet none of the differences achieved statistical significance. While it is tempting to speculate that these variations in SSA might represent biological variation, more likely this reflects methodological variation since SSA is a reactive aldehyde that may be partially lost prior to analytical measurement. Nonetheless, the absence of elevated SSA contrasts with the minor increase previously detected in whole brain extracts of Aldh5a1^-/- ^mice [24]. The putative metabolite of GABA (via further oxidative metabolism of SSA; see Fig. 1), DHHA, increased with gestational age for Aldh5a1^-/- ^mice (Fig. 2), and it was significantly increased at all embryonic ages. For DHHA, there was insufficient material available to examine the concentration at E10-13. Guanidinobutyrate (GB), also believed to derive from GABA, showed a significant increase in Aldh5a1^-/- ^mice, but only later in gestation and at birth (Fig. 2).

**Figure 4:**
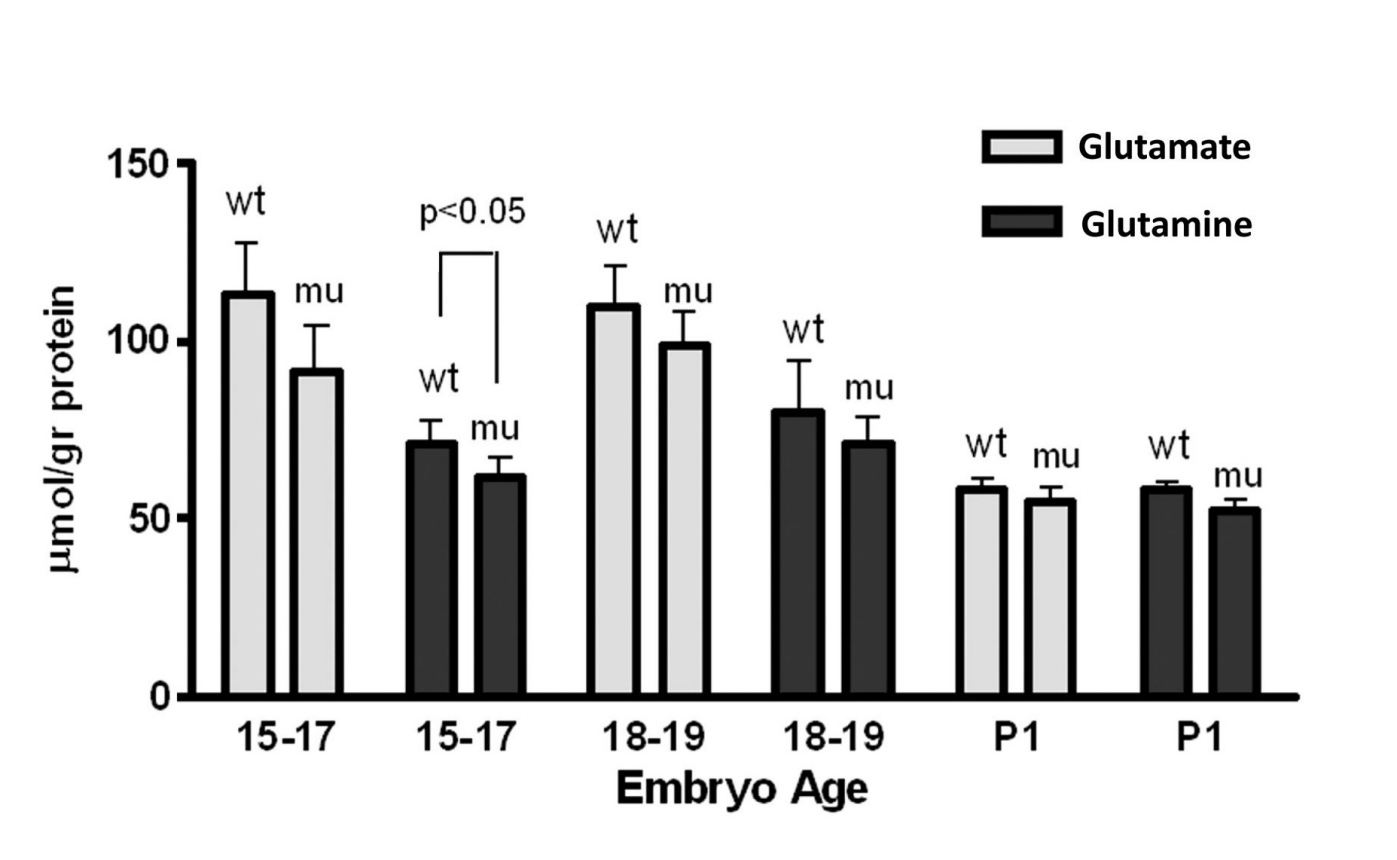
**Concentrations of glutamate and glutamine in embryo extracts as a function of age.** Embryos were grouped as E15–17, 18–19 and day of life 1 (P1). Abbreviations: wt, wild-type (Aldh5a1^+/+ ^mice); mu, mutant (Aldh5a1^-/- ^mice). Statistical analysis performed using a two-tailed t test. One-way ANOVA with Tukey post-hoc analysis revealed a significant decline in glutamate level at birth as compared to E15–17 and E18–19, whereas glutamine levels were not significantly decreased.

GHB was significantly increased with genotype at all embryonic ages (Fig. 3). This increase revealed a linear progression with increasing gestational age, as was the case for the GHB analogue, D-2-hydroxyglutaric acid (D-2-HG; Fig. 1). These data provide further evidence that both species are interrelated in their metabolic sequences. For D-2-HG, the significance at E10–13 was reversed, in that Aldh5a1^+/+ ^mice had significantly larger concentrations than did Aldh5a1^-/- ^mice. The reason for this remains unclear. In the same embryo extracts, we also examined total amino acid patterns to see if particular trends were present. There were no consistently significant differences for any amino acid by genotype, with the exception of GABA. Results for glutamate and glutamine, the direct precursors of GABA, are displayed for E15–17, E18–19 and P1 embryos (Fig. 4). In only a single instance (glutamine, E15–17, p < 0.05), there was no significant difference for either amino acid by genotype, although the trend was for lower levels of both in Aldh5a1^-/- ^mice (Fig. 4). These data are consistent with earlier studies in newborn Aldh5a1^-/- ^mice, in which no differences were observed in glutamine and glutamate by genotype at birth [13,18]. Of note, however, was the considerable decrease in glutamate levels with age. One way ANOVA with Tukey post hoc analysis revealed a significant difference (p < 0.05) for glutamate levels at E15–17 and E18–19 as compared to the same levels at P1 (Fig. 4). This decrease at birth corresponded to the trend toward lower GABA at birth (Fig. 2) for both genotypes.

**Figure 5:**
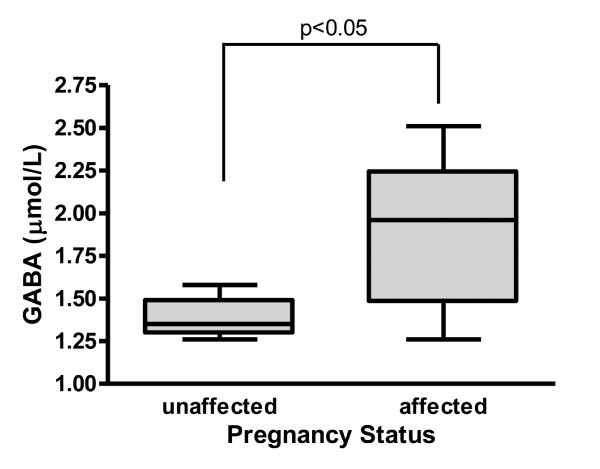
**GABA concentration in amniotic fluid derived from pregnancies carrying an affected SSADH-deficient human fetus.** Data are depicted as box and whiskers, showing the range of values and quartiles. The box extends from the 25th centile to the 75th centile, with a line at the median (50th centile). Whiskers extend above and below the box to show the highest and lowest values. Statistical analysis performed using a two-tailed t test, n = 5 fluid samples for each group. Concentrations of other metabolites (DHHA, D-2-HG and SSA) were not significantly different as a function of pregnancy status.

The finding of elevated embryonic GABA concentrations suggested that amniotic fluid derived from human pregnancies affected with SSADH (Aldh5a1) deficiency might also demonstrate elevated GABA. We have previously shown that an affected pregnancy can be identified by increased GHB levels in amniotic fluid [25]. Accordingly, we examined GABA and other metabolic intermediates in archival amniotic fluid samples for which an SSADH-deficient fetus had been identified. For these fluids from at-risk pregnancies, Aldh5a1-deficiency was documented for the propositus utilizing either enzyme or molecular studies in blood, or both, and a prior affected proband existed in the family. The normal control value for GHB in amniotic fluid was 1.18 ± 0.08 μmol/L (SEM; range 0.42–2.20, n = 31 samples) while the same value for affected amniotic fluids was 5.78 ± 1.25 μmol/L (range 2.10–8.96, n = 5; p < 0.0001). As depicted in Fig. 5, the GABA concentration in these same affected pregnancies was also significantly elevated (1.38 ± 0.05 (SEM), n = 5 for unaffected; 1.88 ± 0.20, n = 5 for affected; p < 0.05). These data imply that GABA is also increased in the SSADH-deficient human fetus, consistent with our results in the murine embryo studies.

## Discussion and conclusion

A cardinal manifestation of SSADH deficiency is hyperintense signals in the globi pallidi bilaterally. This is not unique to this disorder, however, and may relate to ongoing oxidative damage [26]. Nonetheless, this feature is prominent on the MRI of documented patients. We recently identified a 19 year-old patient with SSADH deficiency who, having experienced recurrent seizures for 6 years, died suddenly and unexpectedly [27]. Detailed post-mortem examination revealed striking discoloration of the globi pallidi bilaterally. Accordingly, we hypothesized that the CNS of SSADH-deficient patients might be predisposed to neuroanatomical lesions linked to prenatal exposure to supraphysiological concentrations of GHB, GABA and other intermediates known to accumulate in SSADH deficiency. Our laboratory has previously shown that alterations of myelin formation occur in Aldh5a1^-/- ^mice [28]. We have hypothesized that the metabolite accumulations detected in Aldh5a1^-/- ^mice, including GHB and GABA, alter signaling components (MAP kinase and neurosteroids) that link to use-dependent down-regulation of GABAergic receptors. Other groups have demonstrated that myelin abnormalities can induce oxidant stress [29]. The current report supports our findings of metabolite perturbations in neonatal Aldh5a1^-/- ^mice, and has laid the groundwork for exploration of additional pathophysiological studies in embryonic mice.

GABA is significantly elevated in very early embryonic development of Aldh5a1^-/- ^mice; as well, GHB is similarly increased, suggesting that the enzyme responsible for conversion of SSA to GHB, SSA reductase (aldo-keto reductase 7a2; AKR7a2), is also active in early embryonic development. The linear increase in GHB (Fig. 2) would suggest that AKR7a2 is also demonstrating a developmental ontogeny, which has not been previously documented. Rumigny and colleagues [30] demonstrated that putative AKR7a2 activity was, however, roughly constant in different rodent brain regions for the first two months of life. A similar process occurs for the GHB derivative, D-2-HG (Fig. 2). D-2-HG is derived from GHB in a reaction catalyzed by d-2-hydroxyglutarate transhydrogenase (HOT), an enzyme known to manifest a developmental ontogeny in the rodent [31,32]. The corresponding linear increases for GHB and D-2-HG provides further evidence for the existence of HOT in embryonic rodents, and raises the possibility that D-2-HG may have a pathophysiological role in the developing Aldh5a1^-/- ^embryos, since this compound has been shown to induce oxidative damage in rodent tissues [33-36]. The accumulation of D-2-HG was specific, since there was no increase in isomeric L-2-hydroxyglutarate with any embryological age (data not shown).

Previous studies from our laboratory demonstrated a small, yet significant, increase in total brain SSA levels for Aldh5a1^-/- ^mice [24]; conversely, we saw no evidence for accumulation of SSA in developing embryos. These data suggest that the requisite enzymes which convert SSA either to GHB or DHHA are functional at the developmental time-points we examined. Our data for DHHA, a species unique to Aldh5a1 deficiency, were also revealing. The synthetic pathway for DHHA formation remains to be conclusively demonstrated, but it has been proposed that DHHA derives from condensation of SSA with a 2-carbon species related to pyruvate metabolism (e.g., acetyl-CoA, pyruvate, etc; see Fig. 1) [37]. If this hypothesis is correct, potential disruptions of intermediary metabolism are occurring very early in the development of the Aldh5a1 embryo. These perturbations, especially focused in mitochondrial metabolism, may underlie a component of the imaging abnormalities observed in patients. Additionally, we have shown that DHHA is a weak ligand for the GHB receptor [38], possibly potentiating the excitatory state induced by early GABA and GHB accumulation. GABA-related guanidinobutyrate (GB) reached significant increases in Aldh5a1^-/- ^mice at ~E18–19 (Fig. 2). GB is purported to derive through the catalytic action of the arginine glycine amidinotransferase (AGAT) reaction, which normally produces the creatine precursor guanidinoacetate from arginine and glycine. Substitution of GABA for glycine would lead to production of GB, as has been previously demonstrated [17,39]. Braissant and colleagues [40] demonstrated that AGAT is expressed as early as E12.5 in rat hepatic primordial, and rapidly attains an adult expression pattern thereafter, verifying the importance of creatine formation in the developing embryo. Increased GB in Aldh5a1^-/- ^embryos is consistent with the temporal expression demonstrated by Braissant and colleagues, and suggests that accumulated GABA in these embryos may alter the production of creatine, an important mediator of energy production both in muscle and neural tissue.

During central nervous system (CNS) development, the role of forebrain GABA is switched from an excitatory transmitter to an inhibitory transmitter, the result of an inhibition of calcium influx into postsynaptic neurons derived from the release of GABA. The switch is influenced by the neuronal chloride concentration. Prevailing data suggest that GABA remains excitatory until approximately the first postnatal week in rodents, and that chloride concentrations level out after approximately postnatal week 2. When the neuronal chloride concentration is at a high level, GABA acts as an excitatory neurotransmitter. When neuronal chloride concentration decreases to some degree, GABA acts as an inhibitory neurotransmitter. The neuronal chloride concentration is increased by the Na^+^-K^+^-Cl^-^-cotransporter 1 (NKCC1), and decreased by K^+^-Cl^-^-cotransporter 2 (KCC2) [16,41,42]. As well, GABA is thought to have significant effects on cell migration, differentiation, and synaptogenesis, yet fetal brains of mice lacking both GAD65 and GAD67 have 0.02% of normal GABA content and die at birth, but they have no obvious structural brain abnormalities [15]. Thus, the question posed by our data is whether a marked increase in fetal brain GABA affects cell migration, differentiation, and synaptogenesis or has an effect on the propensity for seizures? Elevation of GHB may play a role on GABA_B_R presynaptically, since the presynaptic GABA_B_R is functional at or around birth, but the postsynaptic GABA_B_R is not functional until about P14. Additionally, the combination of elevated GABA plus GHB would be predicted to have a greater summed effect on GABA_B_R since GHB has no affinity for the GABA_A_R. Another key question is whether elevated GABA in the embryo would alter expression of the cotransporters described above?

Several experiments are underway to address the preceding questions. We are performing pilot studies to evaluate GABA and glutamatergic receptor expression in selected Aldh5a1^-/- ^and Aldh5a1^+/+ ^embryos, in order to test the hypothesis that there is use-dependent down-regulation during development. As well, Western blot studies and real time PCR evaluation of the neuronal ion cotransporters have been planned in conjunction with the preceding evaluations. Studies of axonal and neuronal number and localization have been planned to ascertain if elevations of GHB and GABA have an adverse effect of these parameters during embryo development. Nonetheless, MRI analysis of human Aldh5a1-deficient brain does not provide evidence for structural abnormalities, beyond the characteristic globi pallidi abnormalities observed. Limited neuroimaging in Aldh5a1^-/- ^mice has provided evidence of cerebellar atrophy and some subtle differences in neuronal counts, but more extensive studies are needed [43]. Finally, an emergent approach in our laboratory is to examine methods to deplete elevated GABA and GHB levels in developing embryos. Accordingly, one series of studies in progress is testing the hypothesis that administration of L-histidine to pregnant Aldh5a1^+/- ^dams may drive the formation of homocarnosine from GABA (see Fig. 1) in brain. Whether this has an adverse or positive effect on phenotypic outcome in Aldh5a1^-/- ^mice remains to be determined.

## Authors' contributions

EEWJ and EAS developed isotope dilution methodologies for all assays and performed metabolite measurements on all samples. CJ interpreted all metabolite data, performed statistical analyses on same, and assisted with drafting of the manuscript. EJH oversaw and assisted with all animal husbandry, matings, isolation of embryos, genotyping and other characterization of metabolites. OCS assisted in manuscript development, and oversaw/implemented all amino acid analysis in embryo extracts. KMG helped draft the manuscript, interpreted and analyzed all final metabolic data, and was responsible for development of all figures and final disposition of the paper. Design and coordination of the study was overseen jointly by CJ, OCS and KMG. All authors read and approved the final manuscript.
